# Identifying Risk Profiles for Dementia and brain MRI markers: An Exploratory Analysis of Modifiable Factors in the Memento Cohort

**DOI:** 10.1002/alz70860_098367

**Published:** 2025-12-23

**Authors:** Leslie Grasset, Geneviève Chêne, Carole Dufouil, the MEMENTO Study group

**Affiliations:** ^1^ Univ. Bordeaux, INSERM, BPH, U1219, Bordeaux, France; ^2^ Bordeaux Population Health Research Center, Inserm U1219, University of Bordeaux, Bordeaux, France

## Abstract

**Background:**

As dementia is known to be the consequence of lifelong exposures to a wide range of determinants, an integrative approach is needed to better understand the multifactorial interactions at play leading to pathological brain aging. We propose an exploratory analysis to identify profiles with regard to dementia risk and brain MRI markers.

**Method:**

We used data from 2257 dementia‐free participants of the Memento study, a French nationwide cohort, following patients from 28 memory clinics with either isolated cognitive complaints or mild cognitive impairment. First, we applied latent class analysis to identify clusters of individuals, based on known modifiable protective and risk factors of dementia (e.g. socioeconomic status (SES), vascular risk factors, or lifestyle factors). Then, we investigated the associations between clusters and both dementia risk over 5 years follow‐up using Cox proportional‐hazard models and evolution of brain MRI markers (structural and white matter hyperintensities (WMH)) over 2 years using linear mixed models.

**Results:**

We identified four clusters of risk factors: 1/ “low SES, higher vascular risk, suboptimal lifestyle“ (*n* = 275), 2/ “mixed SES, higher vascular risk, fair lifestyle” (*n* = 635), 3/ “high SES, medium vascular risk, mixed lifestyle” (*n* = 856), and 4/ “intermediate SES, low vascular risk, and healthy lifestyle” (*n* = 491). Compared to cluster 3, cluster 1 was significantly associated with higher risk of dementia (HR_1vs3_=2.02 (1.41–2.91)) and lower total brain and hippocampal volumes at baseline (β_1vs3_=‐16.01 (‐26.59;‐5.43); and ‐0.22 (‐0.36;‐0.08), respectively). Cluster 2 and 4 were non‐significantly associated with higher dementia risk (HR_2vs3_=1.28 (0.97–1.69); HR_4vs3_=1.39 (0.98–1.95)), but were associated with lower dementia risk than cluster 1 (HR_2vs1_=0.63 (0.44–0.91); HR_3vs1_=0.68 (0.46–1.01)). Cluster 2 was associated with lower total brain volumes at baseline than cluster 3 (β_2vs3_=‐8.48 (‐16.14;‐0.86). There was also a non‐significant trend toward lower WMH volume for cluster 4 compared to cluster 3 (β_4vs3_=‐0.09 (‐0.21;0.02)). Results were similar after accounting for APOE‐ε4 status.

**Conclusion:**

This study emphasizes a combination of risk factors, predominantly influenced by socioeconomic status, that drive dementia risk independently of genetic predisposition. Additionally, brain structural volumes are notably impacted by lower SES profiles, whereas cerebrovascular lesions are influenced by vascular risk profile.

